# Prevalence of *Listeria monocytogenes* in Raw Meats Marketed in Bangkok and Characterization of the Isolates by Phenotypic and Molecular Methods

**DOI:** 10.3329/jhpn.v29i1.7565

**Published:** 2011-02

**Authors:** Nitaya Indrawattana, Tanaporn Nibaddhasobon, Nitat Sookrung, Manas Chongsa-nguan, Anchalee Tungtrongchitr, Sou-ichi Makino, Witawat Tungyong, Wanpen Chaicumpa

**Affiliations:** ^1^ Department of Microbiology and Immunology, Faculty of Tropical Medicine, Mahidol University, Bangkok 10400, Thailand; ^2^ Office for Research and Development, Faculty of Medicine Siriraj Hospital, Mahidol University, Bangkok 10700, Thailand; ^3^ Department of Parasitology, Faculty of Medicine Siriraj Hospital, Mahidol University, Bangkok 10700, Thailand; ^4^ Obihiro University of Agriculture and Veterinary Medicine, Hokkaido 080-8555, Japan

**Keywords:** Characterization, Phenotype, Flagellin, Genes typing, Haemolysin, Internalins, Invasins, *Listeria monocytogenes*, Listeriolysin, Listeriosis, Microbial sensitivity tests, Virulence genes, Thailand

## Abstract

*Listeria monocytogenes* causes listeriosis characterized by septicaemia, encephalitis, and abortion or stillbirth. Regular monitoring of its prevalence in food and characterization of its phenotypes and genotypes are necessary for disease surveillance and tracing the epidemic outbreaks. In this study, the prevalence of *L. monocytogenes* in raw meats marketed in Bangkok was 15.4%. The bacteria isolated from meat were serotyped and genotyped using enterobacterial repetitive intergenic consensus–polymerase chain reaction (ERIC-PCR). Their virulence-associated genes, antimicrobial susceptibility, and ability to invade intestinal epithelial cells were studied. All 22 *L. monocytogenes* strains isolated from 104 raw meat samples carried virulence-associated genes, such as *act*A, *fla*A, *hly*A, *iap*, *inl*A, *inl*B, and *prf*A. These were serotype 4b, suggesting their pathogenic and epidemic potential. These isolates could be classified into six ERIC-PCR groups: A-F. The majority (59.1%) of the isolates belonged to Group A, and three isolates were Group D which was closely related to the Group A. Two isolates each were Group C and E, and one isolate each was group B and F. Although the isolates belonged to the same serotype and genotype and were all equipped with the virulence-associated genes, they showed a different cell invasion capability and antibiotic susceptibility. All the isolates were susceptible to ampicillin**,** amikacin, chloramphenicol, gentamicin, imipenem, penicillin G, sulphamethoxazole-trimethoprim, and tetracycline. However, one isolate showed only intermediate susceptibility to tetracycline. The data provide the first molecular insight into the *L. monocytogenes* isolates in Thailand and elucidate a potential risk of people contracting listeriosis.

## INTRODUCTION

*Listeria monocytogenes*, a Gram-positive intracellular bacterium, causes foodborne listeriosis*.* The bacteria can grow under a wide range of temperature and conditions, and these are, thus, ubiquitous (1). *L. monocytogenes* strains attach to environmental surfaces and form biofilms, perhaps by using peritrichous flagella encoded by *fla*A gene (2). The biofilm formation renders the organisms more resistant to harsh environmental conditions, such as food processing, antibiotics, and detergents (3, 4).

Humans are infected by consuming contaminated food. Only a fraction of the infected persons are, however, afflicted while others remain asymptomatic but may harbour the organism in their gastrointestinal tracts (5). The interplay among the host immunity, especially the innate and cell-mediated, the infecting dose, and the virulence of the bacteria regulates the outcome of infection (6). Clinical manifestations of listeriosis range from gastrointestinal disturbances, i.e. non-bloody diarrhoea, nausea, and vomiting, to influenza-like illness with high fever, headache, and myalgia, and to serious septicaemia and meningitis (7). Individuals prone to symptomatic listeriosis include infants, elderly, pregnant women, and those with the underlying conditions which impair their immune functions, such as malnutrition, major surgery, low gastric acidity, and lack of physical fitness (8, 9). Pregnant women infected with *L. monocytogenes* may have spontaneous abortions, stillborn foetuses, or newborns with meningitis (10–12).

Several virulence factors contribute to the pathogenicity of *L. monocytogenes*. The ingested bacteria use several factors, including internalins (Inls), invasion-associated protein encoded by *iap*, murein hydrolase enzyme (p60), surface protein p104, and actA protein encoded by *act*A, to invade the intestinal epithelium via the epithelial tight junction and enter the enterocytes from the lateral surface (13). M cells also endocytose and transport the bacteria to submucosal dendritic cells and macrophages in the dome area of Peyer's patch (14). *L. monocytogenes* uses a pore-forming toxin, i.e. listeriolysin O encoded by hlyA, and caseinolytic proteins (Clp) to digest the phagosomal membrane and free themselves into the cytosol where they multiply extensively (15, 16). The bacteria are disseminated via the lymphatic and blood circulatory systems to several tissues and organs, e.g. liver, spleen, and the central nervous system (17–20). Invasion of the brain results in massive infiltration of inflammatory cells into the tissue causing severe meningitis (21). Intercellular spread of *L. monocytogenes* is mediated by the surface-exposed actA protein which induces the formation of polarized actin filament, along which the bacteria move to the cell membrane to form a bulge-out structure called listeriopod (22, 23). The bacteria are then engulfed by the adjacent cell into a double-membrane vacuole and exit to the cytosol using the listeriolysin O and other phospholipases (24, 25).

Although agriculture and food production are the main occupations of Thai people, and agricultural products are the main export goods of the Kingdom, there is a paucity of information on the characteristics and virulence factors of *L. monocytogenes* which could be isolated from food items. Recently, we made a survey of pathogens in food samples in supermarkets and open markets in Bangkok and its periphery and found a certain prevalence of *L. monocytogenes* contamination (26). In this study, we isolated *L. monocytogenes* from raw meat samples collected from different markets in the Bangkok metropolitan area. We also investigated the pathogenic potential of the isolates by determining their virulence-associated genes. The isolates were genotyped and their ability to invade human epithelial cells and their antibiotic sensitivity were studied. To the best of our knowledge, this is the first study on molecular characterization and genotyping of *L. monocytogenes* reported from Thailand.

## MATERIALS AND METHODS

### Food samples and bacterial isolation

In total, 104 raw meat samples were collected from various supermarkets and open markets in Bangkok during April-November 2007. The samples were transported in clean plastic bags chilled on ice to the laboratory within one hour after sampling. Twenty-five g of each sample was placed into a bag containing 225 mL of Half Fraser's broth (Oxoid, Hampshire, UK). Food homogenization was done using a Stomacher 400 laboratory blender (AES Laboratoire, Bruz, France) for 2-3 minutes, followed by incubation at 25 ºC for 48 hours. Then, 100 µL of each sample were inoculated into 10 mL of Fraser's broth (FB) in a culture tube and incubated at 37 ºC with shaking (250 rpm) for 48 hours. Aliquots (60 µL) of positive FB cultures, i.e. dark colour caused by esculin hydrolysis, were plated individually on BBL CHROM agar and PALCAM agar (Oxoid), and the plates were incubated at 37 ºC for 48 hours. The greenish-black colonies on the PALCAM agar and the blue colonies with a white halo on the BBL CHROM agar were separately subcultured onto tryptone soy agar (TSA) (Oxoid) supplemented with 2% of soy yeast extract (TSYEA) (Oxoid) and incubated at 37 ºC overnight. Esculin-positive colonies were isolated and subjected to Gram-staining and various tests, including CAMP test, catalase, sugar use, nitrate reduction, urease, and motility.

### PCR for detection of virulence gene

Genomic DNA was extracted from the bacterial cells grown at 37 °C overnight in tryptic soy broth (TSB) using a DNA extraction kit (RBC Bioscience, Taipei, Taiwan). Genes encoding actin (*act*A), flagellin (*fla*A), listeriolysin (*hly*A), invasion-associated protein (*iap*), internalin A (*inl*A), internalin B (*inl*B), and pleiotropic activator of virulence genes (*prf*A) were amplified by the oligonucleotide primer sequences shown in Table 1 (27-31). The PCR mixture (25 µL) consisted of: 1 µM of each primer, 100 ng of DNA template, 2.5 µL of 10×*Taq* PCR buffer, 0.2 mM dNTP, 2 mM MgCl_2_, and 1 unit of *Taq* DNA polymerase (Fermentas, St. Leon-Rot, Germany). The PCR mixture was subjected to the following thermal cycle conditions using the Lifecycler (BioRad, California, USA): five minutes of 95 °C before 30 cycles of amplification at 95 °C for 45 seconds, 60 °C for 45 seconds, and 72 °C for 45 seconds for *act*A, *fla*A, and *iap* respectively, 30 cycles of amplification at 95 °C for 45 seconds, 50 °C for 45 seconds, and 72 °C for 45 seconds for *hly*A; and 35 cycles of amplification at 95 °C for 45 seconds, 58 °C for 45 seconds, and 72 °C for 45 seconds for *inl*A*, inl*B, and *prf*A respectively, with a final extension at 72 °C for eight minutes using the Lifecycler (BioRad). The amplified products were analyzed by 1% agarose gel electrophoresis and ethidium bromide staining. The DNA bands were observed under an ultraviolet (UV) transilluminator (Syngene, Cambridge, England).

**Table 1.** Oligonucleotide primer sequences for amplification of virulence-associated genes of *L. monocytogenes* isolates

Target gene	Primer sequence		Size of PCR product (bp)	Reference no.
*hly*A	Forward	5′-CGCGGATGAATTCGATAG-3′	316	27
	Reverse	5′-GTCATACCCGGGAAATCAATG-3′		
*inl*A	Forward	5′-GCTTTCAGCTGGGCATAAC-3′	458	28
	Reverse	5′-ATTCATTTAGTTCCGCCTGT-3′		
*inl*B	Forward	5′-CATGGGAGAGTAACCCAACC-3′	433	29
	Reverse	5′-GCGGTAACCCCTTTGTCATA-3′		
*prf*A	Forward	5′-AACGGGATAAAACCAAAACCA-3′	469	29
	Reverse	5′-TGCGATGCCACTTGAATATC-3′		
*iap*	Forward	5′-ACAAGCTGCACCTGTTGCAG-3′	131	30
	Reverse	5′-TGACAGCGTGTGTAGTAGCA-3′		
*act*A	Forward	5′-CGCCGCGGAAATTAAAAAAAGA-3′	839	30
	Reverse	5′-ACGAAGGAACCGGGCTGCTAG-3′		
*fla*A	Forward	5′-AGCTCTTAGCTCCATGAGTT-3′	450	31
	Reverse	5′-ACATTGTAGCTAAGGCGACT-3′		

PCR=Polymerase chain reaction

### Determination of biofilm formation

Ability to form biofilm of all 84 *Listeria* isolates, which were positive for *fla*A, was determined using the microtiter plate assay (32). Individual isolates were cultured in brain heart infusion (BHI) broth (Oxoid) at 35 °C until the turbidity reached McFarland no. 0.5. Twenty µL of each log phase culture (approximately 3×10^6^ cfu) was distributed into triplicate wells of 96-well flat-bottomed microplate containing 230 µL of the BHI broth. The plate was incubated for 20 hours. Triplicate wells added individually with 20 µL of the BHI broth served as negative controls in each plate. Turbidity of the culture in each well was determined spectrometrically (TECAN, Männedorf, Switzerland). Thereafter, content of each well was discarded, and all wells were washed five times with sterile distilled water before fixing the well surface with 250 µL of methanol, followed by staining with 150 µL of 1% crystal violet (Merck, Damstadt, Germany) in water for 45 minutes. After thorough washing and air-drying, biofilm fixed on each well surface was solubilized in 200 µL of 95% ethanol, and OD at 595 nm was determined. Ability to form biofilm of the bacteria in each plate was graded into negative (mean OD test ≤ mean OD-negative controls of the plate), weak (mean OD test > mean OD-negative controls of the plate to ≤2 x mean OD-negative controls of the plate), moderate (mean OD test >2 x mean OD-negative controls but ≤4 x mean OD-negative controls of the plate), and strong (mean OD test >4 x mean OD-negative controls of the plate) (32).

### ERIC-PCR

An ERIC-PCR was used for genotyping *L.* *monocytogenes*. The oligonucleotide forward primer sequence was 5′-ATGTAAGCTCCTGGGGATTCAC-3′, and the reverse primer sequence was 5′-AAGTAAGTGACTGGGGTGAGCG-3′ (31). Each 25-µL PCR mixture contained 1 µM of each primer, 100 ng of genomic DNA, 2.5 µL of 10×*Taq* PCR buffer, 0.2 mM dNTP, 2 mM MgCl_2_, and 1 unit of *Taq* DNA polymerase (Fermentas). The mixture was subjected to thermal cycles (Lifecycler) which were five minutes of 95 °C before 30 cycles of amplification at 90 °C for 30 seconds, 50 °C for 30 seconds, and 52 °C for one minute, with a final extension at 72 °C for eight minutes. The PCR products were analyzed by 1.2% agarose gel electrophoresis, stained with ethidium bromide and visualized under a UV transilluminator (Syngene). The ERIC-PCR experiments for all the isolates were repeated four times, and reproducible DNA patterns were obtained. The DNA banding patterns were regarded different in the presence of at least one distinct band while the differences in intensity of the bands were not considered. The DNA banding patterns were analyzed using the Gene Directory Application (version 2.01.00) (© 2000-2008 Synoptics Ltd.). Unweighted pair group method with arithmetic averages (UPGMA) and cluster analysis with Dice correlation method based on 1.0% position tolerance were performed.

### Serotyping of ***L. monocytogenes*** isolates

*Listeria* antisera (Seiken, Denka Seika Co. Ltd., Tokyo, Japan) were used for serotyping the *L. monocytogenes* isolates. The assay was performed following the instructions of the manufacturer with modification. For typing of O antigen, each bacterial isolate was grown on TSA at 35 °C overnight. A portion of the bacterial colony was picked up from the colony periphery on the agar plate by a 10-µL loop and suspended in 1 mL of 0.2% normal saline solution. Ten µL aliquots of the cell suspension were added individually with 20 µL of either OI/II or OV/VI polyvalent antisera on glass slides. After mixing thoroughly, the preparations were observed visually for bacterial clumps. Isolates were then appropriately typed with the monovalent antisera, i.e. OI and OIV or OVI, OVII, OVIII, and OIX. For typing of H antigen, a portion of the bacteria from the rim of a colony grown on motility medium at 30 °C overnight was inoculated into 1 mL TSB and incubated at 30 °C overnight. An equal volume of 1% formalin was added to each culture and mixed gently. H antisera, i.e. A, AB, C, and D, were distributed into wells of a 96-well microtitre plate (20 µL per well). The formalin-fixed cell suspension (200 µL) was added appropriately to the antiserum-containing wells. The plate was agitated gently for two minutes and then incubated at 50-52 °C for one hour before visual observation for H agglutination.

### Antibiotic susceptibility testing

The *L. monocytogenes* isolates were assayed for their antibiotic susceptibility using a disc-diffusion method. Individual isolates were grown in TSB at 37 °C until the turbidity of the log phase bacteria reached McFarland no. 0.5. Each bacterial culture was evenly spread onto the surface of a Muller Hinton agarplate (Oxoid). Standard antibiotic discs (ampicillin, amikacin, cefotaxime, ceftazidime, ceftriaxone, chloramphenicol, gentamicin, imipenem, penicillin G, sulphamethoxazole-trimethoprim, and tetracycline) (Oxoid) were individually applied using a disc dispenser, and the plates were incubated at 37 °C for 48 hours. The size of each inhibition zone was determined and interpreted according to the guidelines of the Clinical Laboratory Standards Institute (CLSI) (33).

### Cell invasion assay

The ability of *L. monocytogenes* to invade cells was investigated (34). Confluent monolayers of Caco-2 cells (human colon carcinoma cell-line) were established in a 24-well tissue culture plate (approximately 5×10^5^ cells/well) containing DMEM (Gibco, New York, NY, USA) supplemented with 10% foetal bovine serum and 40 µg/mL gentamicin at 37 °C in a 5% CO_2_ atmosphere. The monolayers were rinsed twice with phosphate-buffered saline, pH 7.4 (PBS), then inoculated with 100 µL of each bacterial suspension containing 5×10^8^ cfu/mL, i.e. the multiplicity of infection (MOI) was 100:1, and the plate was incubated at 37 °C in a 5% CO_2_ incubator for two hours. The cells were rinsed to remove as many extracellular bacteria as possible and then incubated in DMEM containing gentamicin for 1.5 hours. The cells were rinsed with PBS, and 1 mL of ice-cold 0.5% Triton X-100 in distilled water was added. The number of bacteria was determined by plating an appropriate dilution of the cell lysate on TSA. The invasion index of each bacterial isolate was the cfu number on TSA divided by the number in the original inoculum.

### Statistical analysis

The SPSS software (version 17.0) was used for statistical analysis. The two-sided non-parametric chi-square (χ^2^) test was used for analyzing data sorted by types of meat and market. A probability value (p) of <0.05 was considered significantly different.

## RESULTS

### Prevalence of ***L. monocytogenes***

Sixty-one (58.7%) of the 104 raw meat samples, collected during April-November 2007, were positive for *Listeria* spp. based on the selective media and the biochemical tests. Eighty-four isolates of *Listeria* spp. were recovered from the 61 positive samples. Only 22 isolates gave positive results by CAMP test, implying that these were *L. monocytogenes*. These 22 isolates were obtained from 16 meat samples (5 chicken, 8 pork, and 3 beef), i.e. the prevalence was 15.4%. The prevalence of *L. monocytogenes-*associated contamination in each meat type was five of 48 chicken samples (10.4%) (all 5 samples were collected from open markets), eight of 30 (26.7%) pork samples (7 of 19 from supermarkets and 1 of 11 from open markets), and three of 26 (11.5%) beef samples (all 3 samples were collected from supermarkets). Table 2 gives details on the prevalence of *L. monocytogenes* in chicken, pork and beef samples from supermarkets and open markets and the numbers of *L. monocytogenes* isolates recovered from the 16 positive meat samples. There were six meat samples (3 chicken from open markets, 2 pork, and 1 beef from supermarkets) from which two *L. monocytogenes* isolates were recovered from each sample.

**Table 2.** Prevalence of *Listeria monocytogenes* in meat samples from supermarkets and open markets in Bangkok

Meat	Supermarkets (n=49)		Open markets (n=55)		p value
	No. of positive /total number tested (% of prevalence)	No. of isolates	No. of positive /total number tested (% of prevalence)	No. of isolates	
Chicken (n=48)	0/12 (0)^a^	0	5/36 (13.9) ^b^	8	0.00001
Pork (n=30)	7/19 (36.8)^b^	9	1/11 (9.1) ^c^	1	0.00666
Beef (n=26)	3/18 (16.7)^d^	4	0/8 (0)^a^	0	0.00468
Total samples (104)	10/49 (20.4)^b^	13	6/55 (10.9)^d^	9	0.00000

Twenty-two *L. monocytogenes* isolates were recovered from meat, i.e. 8 chicken (no. 1-8) and 1 pork (no. 22) were from supermarkets; 4 beef (no. 9-12) and 9 pork (no. 13-21) were from open markets. Percentages of prevalence of *L. monocytogenes* in the same type of meat collected from supermarkets and open markets and among different types of meat were compared. Entries with different superscripts (a, b, c, and d) are statistically different at p values of <0.05

### Detection of virulence genes

**Fig. 1.**
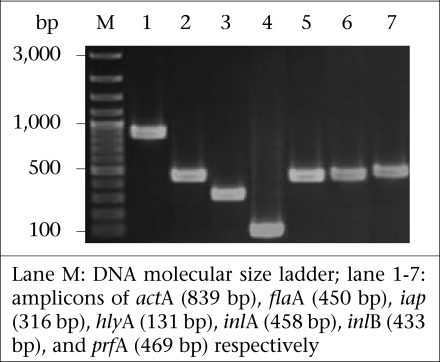
PCR amplicons of 7 virulence-associated genes of L. *monocytogenes* isolates from Bangkok after agarose electrophoresis and ethidium bromide staining

Fig. 1 shows the amplicons of the virulence-associated genes, i.e. *act*A*, fla*A*, hly*A*,* *iap, inl*A*, inl*B*,* and *prf*A*.* Table 3 includes details of the virulence-associated genes of the 84 *Listeria* spp. isolates. The 22 *L. monocytogenes* isolates were positive for all seven genes studied, *i.e.* eight chicken isolates (designated no. 1-8), four beef isolates (no. 9-12), and 10 pork isolates (no. 13-22). The remaining 62 isolates of *Listeria* spp. carried only some seven traits: seven isolates with *act*A, *fla*A, *hly*A*,* and *iap*; 33 isolates with *act*A, *fla*A*,* and *iap*; six isolates with *act*A and *fla*A; 11 isolates with *act*A and *fla*A; three isolates with *fla*A and *iap*; and two isolates with *fla*A only.

**Table 3.** Numbers and prevalence of *L. monocytogenes and Listeria spp.* isolates from meat samples in Bangkok classified by presence of virulence-associated genes that could be amplified from their genomic DNA by PCR and their ability to form biofilm

Bacterial species (no.)	Virulence-associated gene(s)	Ability to form Biofilm			Supermarkets	Open markets
		W***	M	S	No. of positive isolates (% of prevalence)	No. of positive isolates (% of prevalence)
Listeria monocytogenes* (22)	*actA, flaA, hlyA, iap, inlA, inlB, prfA*	3	19		14 (16.7)	8 (9.5)
*Listeria spp.*(7)**	*actA, flaA, hlyA, iap*	6	1		4 (4.8)	3 (3.5)
(33)	*actA, flaA, iap*	17	15	1	12 (14.3)	21 (25)
(6)	actA, flaA, hlyA	5	1		3 (3.6)	3 (3.6)
(11)	*actA, flaA*	7	3	1	7 (8.3)	4 (4.8)
(3)	*oflaA, iap*	2	1		3 (3.6)	0 (0)
(2)	*flaA*	1	1		1 (2.2)	1 (2.2)
Total number of isolates (84)		41	41	2	44 (52.5)	40 (47.5)

*All isolates were haemolytic;

**Non-haemolytic; biofilm formation;

***Number of isolate(s) with indicated ability of biofilm formation; M=Moderate; PCR=Polymerase chain reaction; S=Strong; W=Weak

### Ability to form biofilm

All the 84 isolates formed biofilm, which could be graded into weak (the range of the mean OD_540nm_ was 0.103 to 0.189), moderate (the range of the mean OD_540nm_ 0.200 to 0.388) and strong (the range of the mean OD_540nm_ was 0.423 to 0.503) when compared with the mean OD_540nm_ of negative controls (the range of all the plates was 0.096 to 0.101) (Table 3)

### Serotypes and genotypes of ***L. monocytogenes*** isolates

**Fig. 2.**
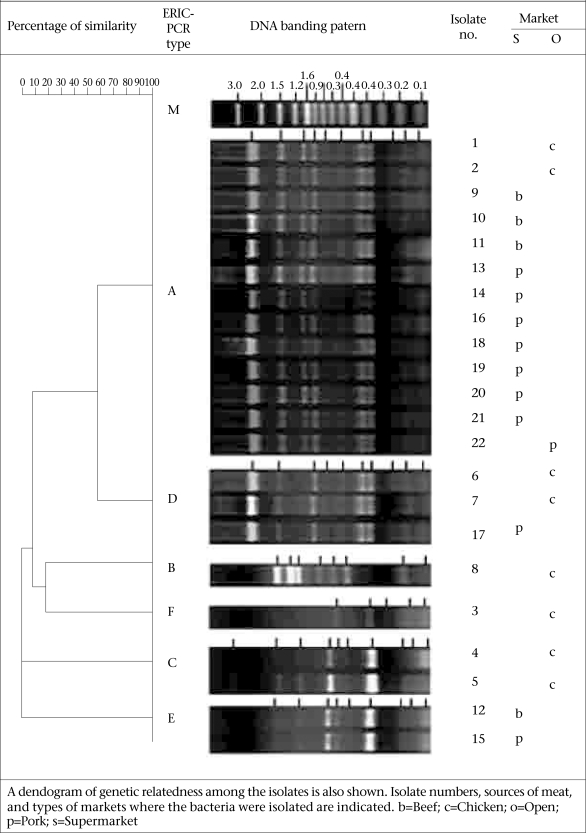
ERIC-PCR DNA fingerprints of 22 L. *monocytogenes* isolates from Bangkok which could be assigned to 6 groups: A-F

The 22 *L. monocytogenes* isolates belonged to the same serotype, i.e. 4b. However, they had different DNA fingerprints by ERIC-PCR, which could be assigned to six groups: A-F (Fig. 2). The majority (59.1%) of the isolates belonged to the ERIC-PCR Group A. Three isolates of Group D were different from Group A by one band (~60% similarity). Group B and F (1 isolate each) showed ~40% similarity to Group A while Group C and E showed a marked diversification from Group A, B, D, and F.

### Ability to invade epithelial cells

**Fig. 3.**
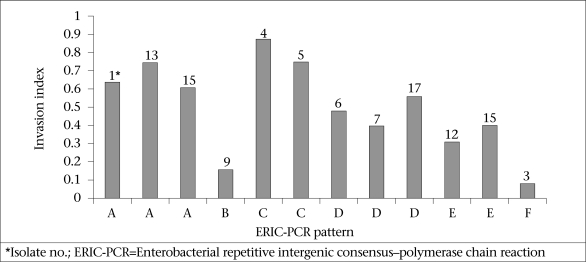
Bar graphs showing invasion indices of L. *monocytogenes* isolates which were positive for all 7 virulence-associated genes and had different ERIC-PCR DNA banding patterns. They were 3 representatives of group A and all isolates of groups B-F respectively.

All the 22 *L. monocytogenes* isolates which were positive for the seven virulence-associated genes readily invaded the Caco-2 cells. Fig. 3 shows the invasion indices of representative *L. monocytogenes* belonging to different ERIC-PCR groups. Isolates belonging to ERIC-PCR Group A and C had the highest invasive capacity, Group D and E had moderate invasiveness, and group B and F revealed the lowest cell invasiveness.

### Antibiotic susceptibility

The 22 *L. monocytogenes* were tested for their antibiotic susceptibility, and the results are presented in Table 4. All the isolates showed a high susceptibility to ampicillin, amikacin, chloramphenicol, gentamicin, imipenem, penicillin G, and the combination of sulphamethoxazole-trimethoprim. One isolate (no. 16) (4.76%) from the pork samples with an ERIC-PCR pattern A showed intermediate susceptibility to tetracycline while the remaining 21 were susceptible to this antibiotic. Most (95.5%) of the 22 *L. monocytogenes* isolates were resistant to cefotaxime, ceftazidime, and ceftriaxone. Nevertheless, one isolate (no. 15) showed intermediate susceptibility to ceftazidime and ceftriaxone (ERIC-PCR pattern E isolated from pork). One isolate from beef with ERIC-PCR pattern A (no. 10) had intermediate susceptibility to ceftazidime while another pattern A isolate (no. 9) was intermediately susceptible to cefotaxime but was susceptible to ceftazidime.

**Table 4.** Antibiotic susceptibility patterns of 22 *L. monocytogenes* isolates

Meat sample	Isolate no.	ERIC-PCR group	Degree of sensitivity to antimicrobial agent										
			AMP	AK	CTX	CAZ	CRO	C	GN	IPM	PG	Te	SXT
Chicken	1	A	S	S	R	R	R	S	S	S	S	S	S
	2	A	S	S	R	R	R	S	S	S	S	S	S
	3	F	S	S	R	R	R	S	S	S	S	S	S
	4	C	S	S	R	R	R	S	S	S	S	S	S
	5	C	S	S	R	R	R	S	S	S	S	S	S
	6	D	S	S	R	R	R	S	S	S	S	S	S
	7	D	S	S	R	R	R	S	S	S	S	S	S
	8	B	S	S	R	R	R	S	S	S	S	S	S
Beef	9	A	S	S	I	S	R	S	S	S	S	S	S
	10	A	S	S	R	I	R	S	S	S	S	S	S
	11	A	S	S	R	R	R	S	S	S	S	S	S
	12	E	S	S	R	R	R	S	S	S	S	S	S
Pork	13	A	S	S	R	R	R	S	S	S	S	S	S
	14	A	S	S	R	R	R	S	S	S	S	S	S
	15	E	S	S	R	I	I	S	S	S	S	S	S
	16	A	S	S	R	R	R	S	S	S	S	I	S
	17	D	S	S	R	R	R	S	S	S	S	S	S
	18	A	S	S	R	R	R	S	S	S	S	S	S
	19	A	S	S	R	R	R	S	S	S	S	S	S
	20	A	S	S	R	R	R	S	S	S	S	S	S
	21	A	S	S	R	R	R	S	S	S	S	S	S
	22	A	S	S	R	R	R	S	S	S	S	S	S

AMP=Ampicillin; AK=Amikacin; C=Chloramphenicol; CAZ=Ceftazidime; CRO=Ceftriaxone; CTX=Cefotaxime; ERIC-PCR=Enterobacterial repetitive intergenic consensus–polymerase chain reaction; GN=Gentamicin; I=Intermediate sensitivity (moderately sensitive); IPM=Imipenem; PG=Penicillin; R=Resistant; S=Highly sensitive; SXT=Sulphamethoxazole-trimethoprim; Te=Tetracycline

## DISCUSSION

*L. monocytogenes* with a gene array coding for virulent-associated factors, including invasins, haemolysin/phospholipases, and actin-polymerizing protein, can cause serious listeriosis which may be fatal (35–37). Although the prevalence of serotypes of *L. monocytogenes* is different in different countries (38–40), serotype 1/2a, 1/2b, 1/2c, and 4b were the ones associated mostly with human listeriosis (41–44). Moreover, the serotype 1/2a, 1/2b, and 1/2c were linked to sporadic cases while the isolates belonging to serotype 4b have been found in patients of listeriosis epidemics (45). Antibiotics, including penicillin, ampicillin, gentamicin, sulphamethoxazole-trimethoprim, and tetracycline have been used for the treatment of human listeriosis (46–48). Regular monitoring of the virulence genes, genotypes, serotypes, and antibiotic susceptibility patterns of the *L. monocytogenes* isolates from humans and environments in a locality is useful for the disease surveillance and effective treatment of the infection. These data were lacking for *L. monocytogenes* isolates in Thailand. However, human listeriosis cases are scarce, and human isolates are not available; we were, thus, interested in looking into the pathogenic potential of food isolates.

In the present study, 15.4% of the raw meat samples collected from supermarkets and open markets in the Bangkok metropolitan area were contaminated with *L. monocytogenes*. In this study, the prevalence was twice higher than data previously reported by our group which was only 7.8% (23/297) among the raw food samples collected from markets in Bangkok and its periphery (different markets from the present study) during June 2006–July 2007 (26). The difference of the *L. monocytogenes* prevalence in the two studies might reflect the different markets of meat sampling and the *L. monocytogenes* identification criteria. Meat in different markets in Bangkok is likely to have different degrees of bacterial contamination due to their different sources and standards of meat processing and handling in individual markets. It has been known that salinity, pH, and water activity (*a*_w_) play an important role in regulating the growth of *L. monocytogenes* (49–51). The bacteria grow better (short generation and lag time) at the alkaline pH and high *a*_w_ (50). At pH of <4.3 and *a*_w_ of <0.930, *L. monocytogenes* survive but do not grow (51). In Thailand, the values of these parameters are not controlled for meats sold in both open- and supermarkets. This might be one of the reasons attributable to the different prevalence of *L. monocytogenes* found in our two studies. Unfortunately, the pH and *a*_w_ of the food samples of both of our studies were not determined. Concerning the identification criteria, isolates which could grow on CHROM and PALCAM agars, gave correct sugar-fermentation patterns, and were positive for *hly*A by PCR were reported as *L. monocytogenes* in our previous study. In the present study, however, the Gram-positive bacteria grown on the two selective agars which gave correct biochemical properties and positive by CAMP test were reported as *L. monocytogenes*. Of the 84 isolates in *Listeria* spp. in this study, 35, i.e. 22 *L. monocytogenes* and 13 *Listeria* spp., carried the gene coding for haemolysin. The presence of *hly*A does not always correlate with the phenotype expression (52). The 13 *Listeria* spp. isolates which were positive for *hlyA* by PCR but non-haemolytic by CAMP test were also negative for *inl*A*, inl*B*,* and *prf*A*.* The remaining 49 non-L*. monocytogenes* isolates harboured only some of the other virulence traits—*act*A*, iap* and/or *fla*A. Thus, the 62 *Listeria* spp. isolates are likely to be non-pathogenic.

Compared to the prevalence of *L. monocytogenes* from raw meat in other geographical areas, the prevalence of *L. monocytogenes* found in the present study was less than the prevalence in other countries, such as raw meat from open markets in Greece and industries and markets in northern Spain, which were 27.5% (beef 20% and chicken 35%) and 34.9% respectively (39, 53). Pork samples were the most frequently *L. monocytogenes*-contaminated meats in this study compared to chicken and beef, which was similar to data reported from elsewhere (54, 55). *L. monocytogenes*-associated contamination was higher in the pork and beef samples from supermarkets than from open markets in Bangkok (p<0.01). In Thailand, meat sold in open markets is supplied daily, usually in the early morning when people buy raw materials for cooking in their home for the day. Meat in supermarkets, especially packed pork and beef, might be left in a refrigerated condition for more than a day during which the contaminating *L. monocytogenes* may readily multiply, causing a higher chance to be detected. Only chicken samples from open markets were contaminated with *L. monocytogenes* in the present study*.* These data conformed to our previous report that chicken samples from open markets were contaminated more than samples from supermarkets (26). In Thailand, the supply of chicken, especially in Bangkok, was from chicken raised in the evaporated house (close) system, and these are processed in poultry processing plants with an export standard. The contamination by *L. monocytogenes* is likely to occur more frequently in open markets than in supermarkets during distribution and handling by retailers.

Molecular insight into the genetic characteristics of the 22 *L. monocytogenes* isolates by determining the presence of virulence-associated genes revealed that all the isolates (100%) carried all the seven traits studied, implying their high pathogenic potential (56). The presence of *flaA* in all the *Listeria* isolates, including the 22 *L. monocytogenes* and the non-*L. monocytogenes*, correlated with their ability to form biofilm which would render the bacteria, more or less, resistant to the harsh environmental conditions and enhance their colonization capability (2–4, 57, 58).

Epidemic tracing of a pathogen, i.e. identification of origin and route of a causative strain in an outbreak, can be efficiently performed by either the phenotypic or genotypic method, or both in combination. For accuracy, however, at least two methods should be used for typing an isolate. In the present study, the *L.* *monocytogenes* isolates were serotyped. All the 22 isolates belonged to serotype 4b; thus, they also had the epidemic potential (59). In our previous report, *L. monocytogenes* isolated from food samples in Bangkok and periphery were serotype 4b (66.67%) and 4c (33.33%) (26). Although the *L. monocytogenes*, contaminated in foods in Bangkok, belonged to the serotype with epidemic potential, outbreak of listeriosis has never occurred, and only a few sporadic cases have been reported occasionally, i.e. two cases with meningitis in 1995 (60) and one case with brain abscess in 2006 (61). The 22 *L. monocytogenes* isolates were also subjected to PCR for detecting seven virulence-associated genes and ERIC-PCR for fingerprints. Their antibiotic susceptibility and ability to invade human epithelial cells were also investigated. Although several methods have been used for genotyping of *L. monocytogenes,* including pulsed-field gel electrophoresis (PFGE), random amplified polymorphic DNA (RAPD), ribotyping, and others, ERIC-PCR was chosen in this study because it has been shown by several investigators to have a high discriminatory power (at least more discriminative than serotyping and phagetyping) and could be performed conveniently (31, 62). Interestingly, the food isolates in this study, which belonged to the same serotype (4b) and carried similarly the seven virulence-associated genes studied, showed diverse DNA banding patterns by ERIC-PCR which emphasized the higher discriminatory accuracy of genotyping compared to serotyping.

Although ERIC-PCR data are difficult to compare between laboratories (because this technique is prone to small differences in assay condition), the ERIC-PCR patterns of the Thai isolates were different arbitrarily from the patterns obtained from *L. monocytogenes* isolated from food samples in Argentina, although the same primers were used in the ERIC-PCR (31). They were also different from food isolates of Bulgaria and Poland (63, 64). Moreover, we found that isolates belonging to different ERIC-PCR groups also had a different ability to invade the Caco-2 cells, although these were equipped similarly with genes coding for cell invasiveness, suggesting the possibility of an unequal expression of the genes by different *L. monocytogenes* isolates. Experiments are needed to elucidate this finding and substantiate our speculation. It was also found that isolates with the same ERIC-PCR patterns were diverse in their antibiotic susceptibility. For example, isolate no. 1, 9, and 10, which had a similar Group A ERIC-PCR pattern, were different in their susceptibility to cefotaxime and ceftazidime, and isolate no. 12 and 15 which had a similar pattern E were different in their susceptibility to ceftriaxone. There was one isolate (4.5%) which was intermediately susceptible to tetracycline (isolate no. 16 from pork). Tetracycline is one of the drugs of choice for the treatment of listeriosis. This might stem from the misuse of this antibiotic as a growth promoter in pigs which provided a selective pressure to the bacteria or it might be due to a horizontal transfer of the antibiotic-resistant gene among the environmental bacteria. A close and regular watch on the antibiotic susceptibility of *L. monocytogenes* is warranted.

Overall, the findings of this study supported the notion of the diversification of *L. monocytogenes* isolated from different geographical areas and emphasized the need of identification, differentiation, and characterization of *Listeria* spp. contaminated in foods to determine their pathogenic and epidemic potential and sensitivity to chemotherapeutic agents. The findings also, for the first time, gave insight into the phenotypic and genetic characteristics and the pathogenic potential of *L. monocytogenes* isolated from foods in Thailand.

## ACKNOWLEDGEMENTS

This work was supported by the DPG5380001 grant of the Thailand Research Fund (TRF) and the National Research University grant from Commission on Higher Education (CHE) through the Center for Biopharmaceutical Development and Innovative Therapy, Mahidol University, Thailand. Nitaya Indrawattana, Nitat Sookrung, and Anchalee Tungtrongchitr are the TRF scholars.
